# Fabrication and Characterization of Porous MgAl_2_O_4_ Ceramics via a Novel Aqueous Gel-Casting Process

**DOI:** 10.3390/ma10121376

**Published:** 2017-11-30

**Authors:** Lei Yuan, Zongquan Liu, Zhenli Liu, Xiao He, Beiyue Ma, Qiang Zhu, Jingkun Yu

**Affiliations:** 1 School of Metallurgy, Northeastern University, Shenyang 110819, China; liuzongspring@163.com (Z.L.); 18842395468@163.com (Z.L.); khuntoriahx@sina.com (X.H.); quainty@126.com (J.Y.); 2 Electron Microscope Unit, Mark Wainwright Analytical Centre, The University of New South Wales, Sydney, NSW 2032, Australia; qiang.zhu@unsw.edu.au

**Keywords:** porous ceramics, MgAl_2_O_4_, hydratable alumina, gel-casting, aqueous

## Abstract

A novel and aqueous gel-casting process has been successfully developed to fabricate porous MgAl_2_O_4_ ceramics by using hydratable alumina and MgO powders as raw materials and deionized water as hydration agent. The effects of different amounts of deionized water on the hydration properties, apparent porosity, bulk density, microstructure, pore size distribution and compressive strength of the samples were investigated. The results indicated that the porosity and the microstructure of porous MgAl_2_O_4_ ceramics were governed by the amounts of deionized water added. The porous structure was formed by the liberation of physisorbed water and the decomposition of hydration products such as bayerite, brucite and boehmite. After determining the addition amounts of deionized water, the fabricated porous MgAl_2_O_4_ ceramics had a high apparent porosity (52.5–65.8%), a small average pore size structure (around 1–3 μm) and a relatively high compressive strength (12–28 MPa). The novel aqueous gel-casting process with easy access is expected to be a promising candidate for the preparation of Al_2_O_3_-based porous ceramics.

## 1. Introduction

Porous MgAl_2_O_4_ spinel ceramics have attracted a growing interest due to their high melting point (2135 °C), good thermal shock resistance, excellent chemical inertness, and their low thermal conductivity and thermal expansion coefficient; they are widely used for various applications such as gas filters, thermal insulation materials, waste water filters, catalyst supports and separation membranes [1,2,3,4,5]. In order to meet specific requirements of porosity and pore sizes for different applications, various available methods have been developed in recent years, including gel casting, the addition of pore-forming agent, sacrificial templating, in-situ synthesis, and combustion synthesis [6,7,8,9,10]. Among these, gel casting has been regarded as one of the most useful methods for fabricating porous MgAl_2_O_4_ ceramics, because it is feasible to obtain materials with complex shapes. However, large volumes of organic compounds are used, and the protection of nitrogen is required during the gel-casting process [11,12]. These organic compounds include monomers, catalysts, initiators and cross-linking agents, which are usually poisonous.

In order to overcome these problems, researchers have developed some less- or non-toxic organic compounds for use as gelling agents, including epoxy resin, gelatin, protein, *N*,*N*-dimethylacrylamide (DMAA) and carboxymethyl cellulose [13,14,15,16,17,18]. The results of this research have been extremely valuable for the development of the gel-casting process, aside from a few small disadvantages. For example, these gelling agents are also organic compounds, which will release CO_2_ or hydrocarbon gases during the heating process of green bodies. Meanwhile, the cost of organic compounds is expensive, and the process of gel casting is relatively complex. Therefore, an absolutely aqueous and more feasible gel-casting process without any organic compounds is desirable for the fabrication of porous MgAl_2_O_4_ ceramics.

Hydratable alumina (ρ-Al_2_O_3_) is a transition alumina, which is generally produced from flash calcination of gibbsite. After contact with water, ρ-Al_2_O_3_ can undergo hydration to generate binding ability [19,20,21]. During the hydration process, firstly, a large amount of gel is formed; and then part of the gel is transformed as bayerite (Al(OH)_3_), with small amounts of boehmite (AlO(OH)) crystals. Simultaneously, the other gel is present as boehmite gel, ultimately accompanied by amorphous gel [19]. The binding strength of the green body is determined by the interlocking bayerite crystal and gel [22]. As a result, ρ-Al_2_O_3_ is always used as a binder in cement-free refractory castables [23,24,25].

Thereby, in combination with the gel-casting process, when introducing ρ-Al_2_O_3_ as the gelling source, it is expected that a novel method for fabricating porous ceramics will result. This is because the hydration of ρ-Al_2_O_3_ can form gels to bind the green body, and small amounts of water can also be enclosed in the green body by the interlocking gels. Subsequently, after drying and heating, a porous structure is obtained due to the liberation of physisorbed water and the ultimate decomposition of the gels. Hence, an absolutely aqueous gel-casting process will be developed for preparing porous ceramics. However, to our knowledge, there have been limited reports on the fabrication of porous ceramics using ρ-Al_2_O_3_ as a gelling source so far [26].

In the current study, an aqueous gel-casting process has been successfully developed for fabricating porous MgAl_2_O_4_ ceramic by utilizing ρ-Al_2_O_3_ and MgO powders as raw materials and deionized water as a hydration agent. The presence of MgO powder can accelerate the hydration of ρ-Al_2_O_3_ to enhance the strength of the green body [27,28]. Furthermore, the porosity of the as-fabricated porous MgAl_2_O_4_ ceramics can be controlled by the amounts of deionized water added. Therefore, the effects of ρ-Al_2_O_3_ and the amount of deionized water on the properties of porous MgAl_2_O_4_ ceramic, in terms of hydration properties, phase composition, porosity, microstructure, pores size and compressive strength, have been investigated and discussed in detail.

## 2. Experimental

### 2.1. Raw Materials and Preparation Process

Commercial ρ-Al_2_O_3_ (Purity ≥ 90%, LOI (Loss on ignition) ≤ 10%, d_50_ ≤ 1 μm, Zhengzhou Non-ferrous Metals Research Institute Co., Ltd., Zhengzhou, China) and MgO powders (Purity ≥ 99%, d_50_ ≤ 1 μm, Sinopharm Chemical Reagent Co., Ltd., Shanghai, China) were used as the starting raw materials. Deionized water was used as hydration agent.

In order to form stoichiometric MgAl_2_O_4_ in the as-fabricated porous ceramics, and in consideration of the purity of ρ-Al_2_O_3_, the weight ratio of MgO and ρ-Al_2_O_3_ was chosen as 1:2.85. The weight ratios of raw materials of samples are listed in Table 1.

Various amounts of MgO and ρ-Al_2_O_3_ were initially fully mixed as the starting powders. Then, the deionized water was added to the starting powders to prepare aqueous slurry. The weight ratios of the starting powders and deionized water were 1:1 (S1), 1:1.5 (S2), 1:2 (S3) and 1:2.5 (S4), respectively. Vigorous mechanical stirring and ultrasonic shaking (Frequency of 40 KHz) were applied for 30 min to guarantee uniform dispersion. After degassing under vacuum conditions for 10 min, the prepared gels were placed into cylindrical molds (φ27 mm) and cured at 25 °C with relative humidity of 85% for 24 h. Finally, after drying at room temperature for 72 h, the green bodies (about φ25 mm) were removed from molds, and subsequently sintered at 1600 °C for 4 h with a heating rate of 1 °C/min over the range from room temperature to 600 °C, and at 4 °C/min from 600 °C to 1600 °C.

### 2.2. Characterization

Apparent porosity and bulk density were determined by the Archimedes method using water as the medium. All characterizations were performed in triplicate. Thermogravimetry and differential scanning calorimeter (TG-DSC) curves were measured by simultaneous thermal analyzer (STA449F3, NETZSCH, Waldkraiburg, Germany) at a heating rate of 4 °C/min. The phase compositions of the dried green bodies and as-fabricated porous ceramics were identified by X-ray diffraction (XRD, Model D500, Siemens, Munich, Germany) using Cu Kα radiation. The microstructure and morphology of the porous ceramics were observed by field emission scanning electron microscope (FE-SEM, Model Ultra Plus, ZEISS, Oberkochen, Germany) equipped with an energy dispersive spectroscopy (EDS, Oxford, UK) unit. The pore size distribution of the samples was examined by mercury porosimetry (Autopore IV9500, Micromeritics Instrument Corp., Norcross, GA, USA). Compressive strength was measured by using hydraulic press machine (5015 type, Shijin Corp., Jinan, China) according to GB/T 5072-2008 at room temperature.

## 3. Results and Discussion

### 3.1. Hydration Properties

**Figure 1:**
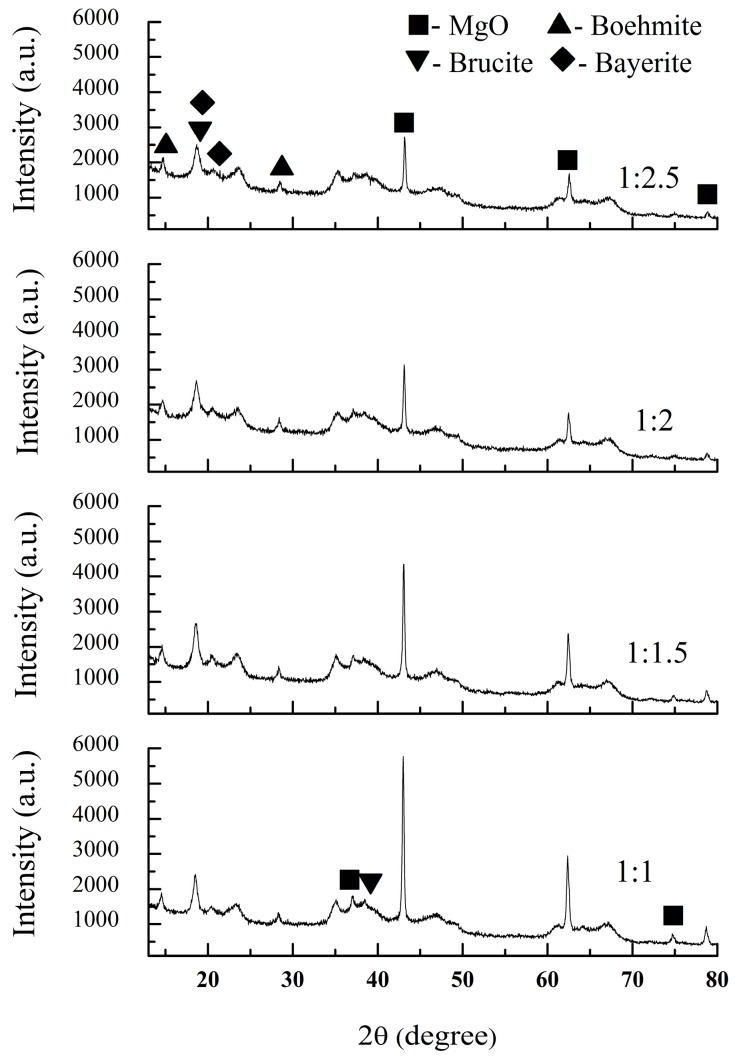
XRD patterns of the dried green bodies with different ratios of starting powders and deionized water: S1 (1:1); S2 (1:1.5); S3 (1:2) and S4 (1:2.5).

Figure 1 shows XRD patterns of the dried green bodies with different ratios of starting powders and deionized water.

In the initial raw materials, ρ-Al_2_O_3_ is amorphous phase and MgO is periclase phase. After adding deionized water to form gels and casting, the phase composition of the green bodies was obviously changed. It can be seen that there were MgO (Ref. code: 01-075-1525), brucite (Mg(OH)_2_, Ref. code: 00-001-1169), boehmite (Ref. code: 01-083-2384) and bayerite (Ref. code: 01-077-0250) phases in all of the samples. That is to say, hydration reactions had occurred in the gel-casting process. When the ratio of starting powders and deionized water was 1:1, ρ-Al_2_O_3_ reacted with water to form boehmite and bayerite, and a certain amount of MgO to form brucite, as shown in reactions (1) and (2), respectively.

ρ-Al_2_O_3_ + 2H_2_O → AlO(OH) + Al(OH)_3_ (1)

MgO + H_2_O → Mg(OH)_2_ (2)

With an increase in the amount of deionized water added, the relative intensity of the MgO peak decreased obviously. This indicates that the amounts of hydration products like Mg(OH)_2_ increased gradually. The intensity of brucite, boehmite and bayerite peaks did not obviously increase. The main reason for this was that the crystallization degrees of brucite, boehmite and bayerite were low, resulting in the relatively low intensity of the peaks.

**Figure 2:**
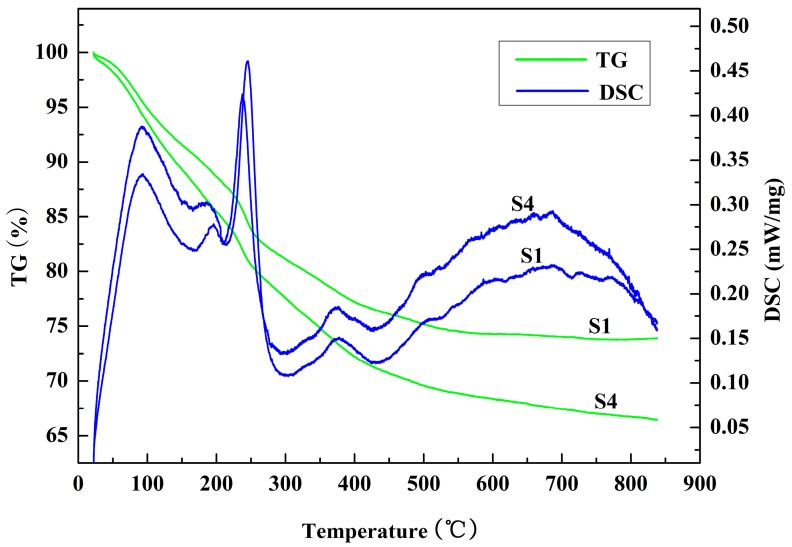
TG-DSC curves of the dried green bodies with different ratios of starting powders and deionized water: S1 (1:1) and S4 (1:2.5).

Figure 2 compares the TG-DSC curves of the dried green bodies with ratios of starting powders and deionized water of 1:1 and 1:2.5. The resulting TG curves show that the weight losses were about 26.2% and 33.3% for samples S1 and S4, respectively. This can be attributed to the liberation of physisorbed water and the decomposition of boehmite, brucite and bayerite. The reason for the higher weight loss of S4 than of S1 was that greater amounts of physisorbed water and hydration products were formed because of the addition of greater amounts of deionized water. After calculation on the basis of reactions (1) and (2), the theoretical weight gain of the samples after hydration was 37.82%. The weight losses of all the samples were lower than the theoretical weight gains of the hydration reactions. This indicates that the starting powders were hydrated incompletely. These results are also consistent with the XRD results in Figure 1.

It can be seen from the DSC curves that there are endothermic peaks appearing at around 90 °C, 185 °C, 250 °C, 375 °C and 500 °C in both sample S1 and S4. The endothermic peaks of 90 °C and 185 °C can be attributed to the liberation of physisorbed water and the decomposition of pseudo-boehmite, respectively [19]. As the heating temperature increased, the decomposition of bayerite and brucite occurred at about 250 °C and 375 °C, respectively. In addition, the weak endothermic peaks at about 500 °C can be explained by the decomposition of crystalline boehmite. These results prove that physisorbed water and the hydration products of brucite, boehmite and bayerite were present in the green bodies. Simultaneously, the intensity of the endothermic peaks in sample S4 are obviously higher than those of sample S1. An explanation for this could be that a higher degree of hydration occurred, forming greater amounts of hydration products in sample S4.

### 3.2. Phase Composition and Sintering Behaviors

**Figure 3:**
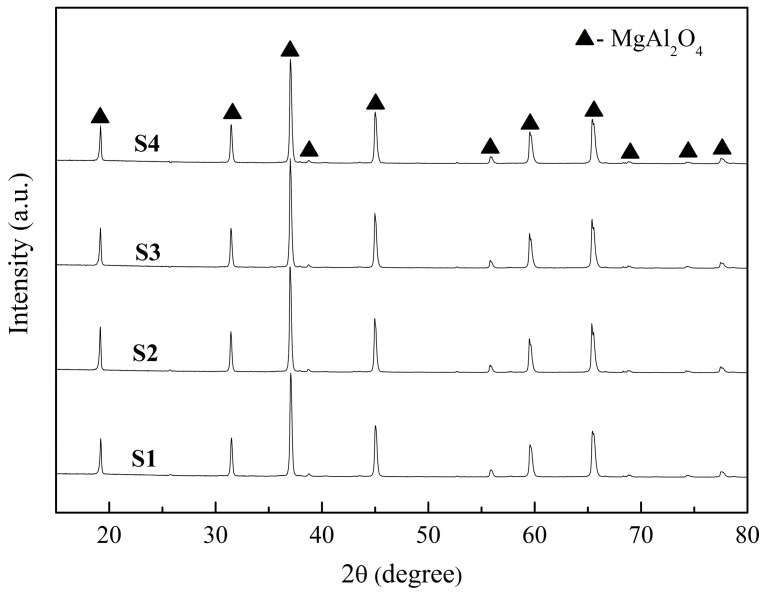
XRD patterns of the samples with different ratios of starting powders and deionized water sintered at 1600 °C for 4 h.

Figure 3 shows XRD patterns of the samples with different ratios of starting powders and deionized water sintered at 1600 °C for 4 h. It can be observed that only MgAl_2_O_4_ spinel phases were detected in all the samples. In this case, the following chemical reactions were likely to have occurred during the sintering process.

2Al(OH)_3_ = Al_2_O_3_ + 3H_2_O (3)

2AlO(OH) = Al_2_O_3_ + H_2_O (4)

Mg(OH)_2_ = MgO + H_2_O (5)

Al_2_O_3_ + MgO = MgAl_2_O_4_ (6)

When the green bodies were submitted to sintering, the hydration products were firstly decomposed, and subsequently formed MgAl_2_O_4_. Meanwhile, a large number of pores formed in the MgAl_2_O_4_ ceramics.

**Figure 4:**
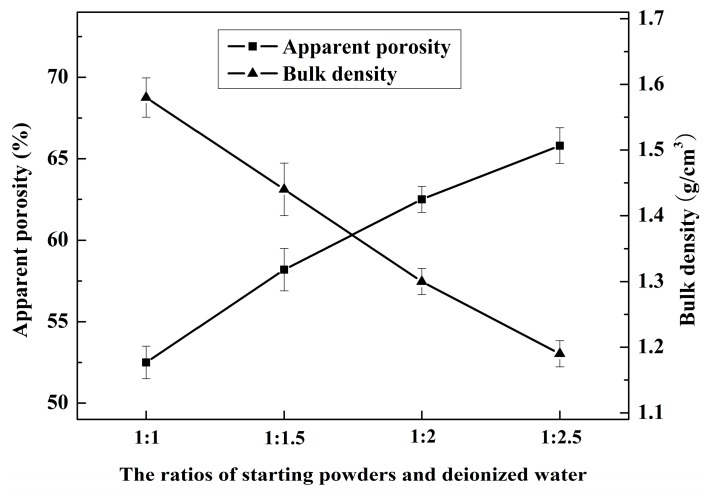
The apparent porosity and bulk density of the samples with different ratios of starting powders and deionized water sintered at 1600 °C for 4 h.

The apparent porosity and bulk density of the samples with different ratios of starting powders and deionized water sintered at 1600 °C for 4 h are shown in Figure 4. It can be seen that the apparent porosity of the as-fabricated porous MgAl_2_O_4_ ceramics reached a range of about 52.5% to 65.8%. The apparent porosity of all of the samples increased, and bulk density decreased markedly with increased addition of deionized water. For the sample with a ratio of starting powders to deionized water of 1:2.5, the apparent porosity was 65.8%, and bulk density reached 1.19 g/cm^3^. This indicates that the physisorbed water and the extent of hydration caused by the deionized water were the main contributors to the apparent porosity. The greater the amount of deionized water that was added, the more pores were able to be constructed by the liberation of physisorbed water and the decomposition of hydration products, resulting in a higher apparent porosity. That is to say, the deionized water is not only a hydration agent, but also a pore-forming agent. However, the continuous addition of even greater amounts of deionized water to the sample was meaningless; for example, the weight percent of addition deionized water was more than 71.4%, because excess water was likely to float on the top of gels during the curing process, resulting in its having an insignificant influence on the apparent porosity of the samples.

### 3.3. Microstructure and Pore Size Distribution

**Figure 5:**
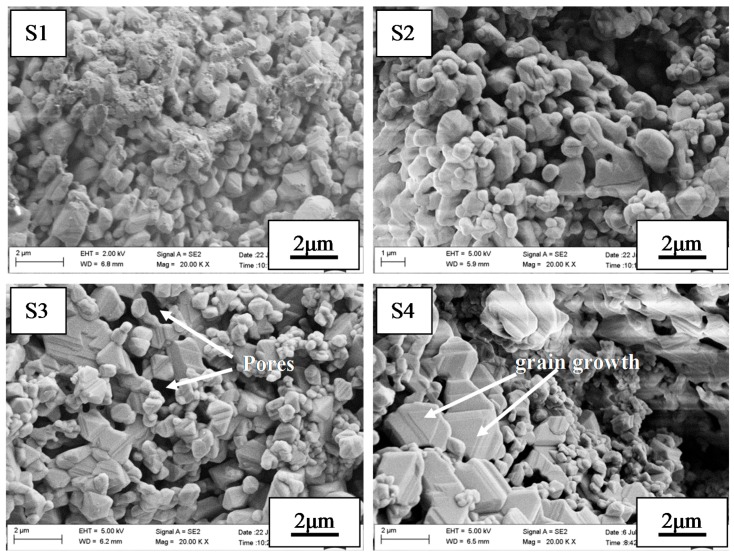
SEM images of fracture surface of the samples with different ratios of starting powders and deionized water sintered at 1600 °C for 4 h: (S1) 1:1; (S2) 1:1.5; (S3) 1:2; (S4) 1:2.5.

Figure 5 shows the SEM images of the fracture surfaces of the samples of as-fabricated porous MgAl_2_O_4_ ceramics with different ratios of starting powders and deionized water, sintered at 1600 °C for 4 h. It can be seen that a porous structure was constructed by MgAl_2_O_4_ crystalline grains interlaced with each other. The porous structure was formed by two aspects: on one hand, the reaction between Al_2_O_3_ and MgO generated MgAl_2_O_4_ spinel accompanying a volume expansion of about 7% volume expansion [29]; on the other hand, the liberation of physisorbed water and the decomposition of the hydration products left a large number of pores during the sintering process. Moreover, with an increase in the amount of deionized water added, the bonding among MgAl_2_O_4_ grains was incompact, resulting in a higher apparent porosity, as shown in Figure 5 (S3,S4). In the case of the sample with a 1:1 ratio of starting powders to deionized water, the grain size of MgAl_2_O_4_ was about 0.5 μm. However, with an increased in the amount of deionized water added, the grain size of part amounts of MgAl_2_O_4_ increased obviously, reaching about 2 μm. This could be attributed to the fact that the MgO and Al_2_O_3_ that had been decomposed by the hydration products promoted the sintering activity of the green bodies. Many researchers have reported that the MgO from produced Mg(OH)_2_ and the Al_2_O_3_ produced from Al(OH)_3_ or AlO(OH) have high activity, high surface area and low activation energy, resulting in a high sintering activity [30,31,32,33]. In the current case, with the amounts of deionized water increasing, the amounts of hydration products (brucite, boehmite and bayerite) increased. That is to say, a greater amount of active MgO and Al_2_O_3_ could be obtained, resulting in a high sintering activity and the easy growth of a small amount of MgAl_2_O_4_ crystalline grains.

**Figure 6:**
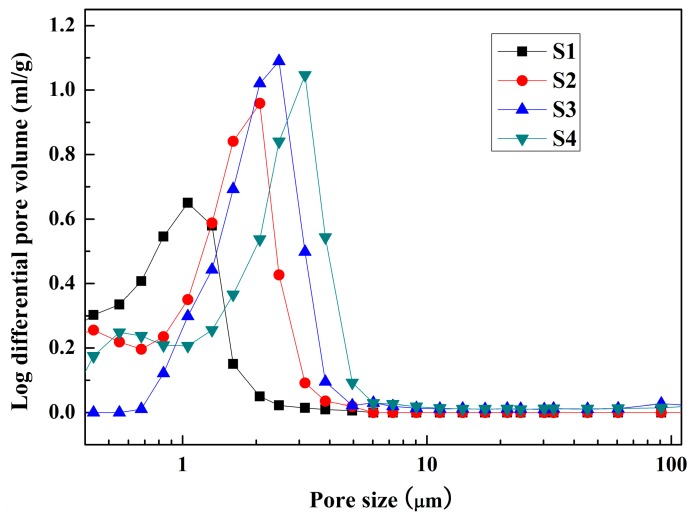
The pore size distribution of the samples with different ratios of starting powders and deionized water sintered at 1600 °C for 4 h.

Figure 6 compares the pore size distribution of the as-fabricated porous MgAl_2_O_4_ ceramics with different ratios of starting powders and deionized water. It can be seen that the average pore size of all the samples is in the range of about 1 μm to 3 μm. This means that the small pore size structure of porous MgAl_2_O_4_ ceramics can be obtained using the novel aqueous gel-casting process. This is attributed to the fact that the pores of the samples were mainly formed by the liberation of physisorbed water and the decomposition of hydration products such as bayerite, boehmite and brucite. Moreover, it can be seen that the average pore size of the samples increased with the addition of increasing amounts of deionized water. The main reason for this was that the increased amounts of physisorbed water in the green body promoted the increase in pore size. Additionally, the growth of a small amount of MgAl_2_O_4_ crystalline grains led to the elimination of fine pores. These results are consistent with the microstructure of the samples. As a result, the amounts of deionized water added not only controlled the porosity, but also effected the pore size distribution of the as-fabricated porous MgAl_2_O_4_ ceramics.

### 3.4. Mechanical Properties

**Figure 7:**
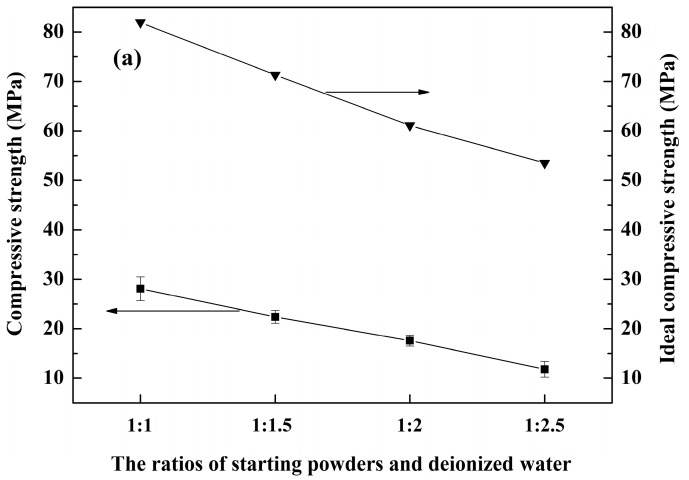

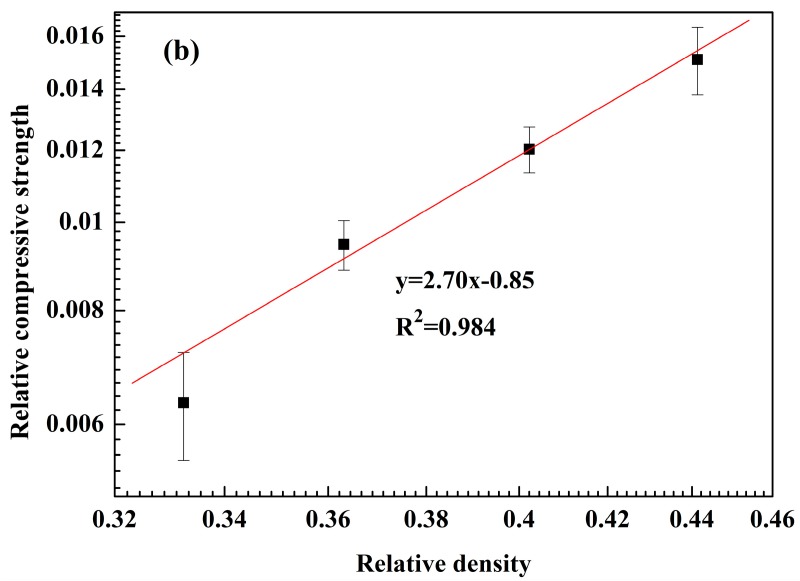
(**a**) Compressive strength and ideal compressive strength of the samples with different ratios of starting powders and deionized water; and (**b**) linear fitting of the relative compressive strength and relative density.

The effects of different ratios of starting powders and deionized water on the compressive strength of the as-fabricated porous MgAl_2_O_4_ ceramics are shown in Figure 7a. It can be seen that a relatively high compressive strength (12–28 MPa) was obtained in the samples. This is attributed to the fact that MgO and Al_2_O_3_ produced from the hydration products promoted the sintering activity in the samples, leading to a tight bond among the crystalline grains. In addition, with an increase in the amount of deionized water added, the compressive strength of the porous MgAl_2_O_4_ ceramics decreased markedly. This could be explained by the fact that the porosity of samples increased with increasing amounts of added deionized water. The relation between bulk density (*ρ_b_*) and compressive strength (*σ*) of a porous ceramic can be expressed according to the Gibson and Ashby model, as described in [34].

*σ* = *σ_s_*·C·(*ρ_b_*/*ρ_s_*)^n^ (7)

where, *σ_s_* and *ρ_s_* are the strength and density of the dense ceramic, respectively. The values of *σ_s_* and *ρ_s_* are 1862 MPa and 3.58 g/cm^3^, respectively, for the current case of dense MgAl_2_O_4_ ceramics. C and n are constants with values of 0.15 and 1.5, respectively, assuming an ideal pore structure [35].

After calculating, the ideal compressive strength of the porous MgAl_2_O_4_ ceramic is shown in Figure 7a. It can be seen that the compressive strength of the present case is lower than that of the ideal strength. Subsequently, after linear fitting of the actual relative compressive strength and density with Equation (7), which is given in Figure 7b, the values of C and n were found to be 0.14 and 2.70, in the current case, respectively. In comparison with the ideal model, the different values of C and n in the present as-fabricated porous ceramics can be attributed to the different pore morphologies and pore sizes. This indicates that the strength of the MgAl_2_O_4_ porous ceramic could be further improved by optimizing its pore size and pore size distribution. However, the compressive strength was still about 12 MPa, even in the sample with a starting powder to deionized water ratio of 1:2.5. This means that the compressive strength of the as-fabricated porous MgAl_2_O_4_ ceramics is able to meet the strength requirements for most applications.

## 4. Conclusions

(1) A novel aqueous gel-casting process was successfully developed to fabricate porous MgAl_2_O_4_ ceramics utilizing ρ-Al_2_O_3_ and MgO powders as raw materials, and deionized water as hydration agent. A high-porosity structure with a small pore size was constructed by the fine MgAl_2_O_4_ crystalline grains.

(2) The amounts of deionized water added were the main factor governing the porosity and microstructure of the as-fabricated porous MgAl_2_O_4_ ceramics.

(3) With increasing the amounts of deionized water added, the apparent porosity of the as-fabricated porous MgAl_2_O_4_ ceramics increased markedly, and the bulk density and the compressive strength decreased. When the ratio of starting powders and deionized water was 1:2.5, the apparent porosity reached 65.8% and the compressive strength was 12 MPa.

## Figures and Tables

**Table 1:** Ratios of raw materials of the samples (wt %).

Samples	Starting Powders		Deionized Water
	ρ-Al_2_O_3_	MgO	
S1	37.0	13.0	50.0
S2	29.6	10.4	60.0
S3	24.6	8.7	66.7
S4	21.2	7.4	71.4
